# Transcriptome profiling of *Zymomonas mobilis* under ethanol stress

**DOI:** 10.1186/1754-6834-5-75

**Published:** 2012-10-11

**Authors:** Ming-xiong He, Bo Wu, Zong-xia Shui, Qi-chun Hu, Wen-guo Wang, Fu-rong Tan, Xiao-yu Tang, Qi-li Zhu, Ke Pan, Qing Li, Xiao-hong Su

**Affiliations:** 1 Biogas Institute of Ministry of Agriculture, Biomass Energy Technology Research Centre, Section 4-13, Renming Nanlu, Chengdu 610041, China; 2 Key Laboratory of Development and Application of Rural Renewable Energy, Ministry of Agriculture, Chengdu 610041, P. R. China

## Abstract

**Background:**

High tolerance to ethanol is a desirable characteristics for ethanologenic strains used in industrial ethanol fermentation. A deeper understanding of the molecular mechanisms underlying ethanologenic strains tolerance of ethanol stress may guide the design of rational strategies to increase process performance in industrial alcoholic production. Many extensive studies have been performed in *Saccharomyces cerevisiae* and *Escherichia coli*. However, the physiological basis and genetic mechanisms involved in ethanol tolerance for *Zymomonas mobilis* are poorly understood on genomic level. To identify the genes required for tolerance to ethanol, microarray technology was used to investigate the transcriptome profiling of the ethanologenic *Z. mobilis* in response to ethanol stress.

**Results:**

We successfully identified 127 genes which were differentially expressed in response to ethanol. Ethanol up- or down-regulated genes related to cell wall/membrane biogenesis, metabolism, and transcription. These genes were classified as being involved in a wide range of cellular processes including carbohydrate metabolism, cell wall/membrane biogenesis, respiratory chain, terpenoid biosynthesis, DNA replication, DNA recombination, DNA repair, transport, transcriptional regulation, some universal stress response, etc.

**Conclusion:**

In this study, genome-wide transcriptional responses to ethanol were investigated for the first time in *Z. mobilis* using microarray analysis.Our results revealed that ethanol had effects on multiple aspects of cellular metabolism at the transcriptional level and that membrane might play important roles in response to ethanol. Although the molecular mechanism involved in tolerance and adaptation of ethanologenic strains to ethanol is still unclear, this research has provided insights into molecular response to ethanol in *Z. mobilis*. These data will also be helpful to construct more ethanol resistant strains for cellulosic ethanol production in the future.

## Introduction

High tolerance to ethanol is a desirable characteristics for ethanologenic strains used in industrial ethanol fermentation. However, ethanol is generally toxic to microorganisms (such as *Saccharomyces cerevisiae*, *Escherichia coli*, and *Zymomonas mobilis*, etc.), and intracellular and extracellular accumulation of ethanol also inhibits cell growth and metabolism [1-3]. So, ethanol can become a significant stress factor during bio-ethanol fermentation. A deeper understanding of the molecular mechanisms underlying ethanologenic strains tolerance of ethanol stress may guide the design of rational strategies to increase process performance in industrial alcoholic fermentation.

Currently, the mechanism of ethanol stress in *S. cerevisiae* have been studied intensively on cell viability and growth [4], metabolism, cell structure and membrane function [3]. Other studies on transcriptional level also revealed that many genes were more highly expressed in *S. cerevisiae* during ethanol stress, such as heat shock proteins [5]. Alper *et al.* found that a global transcription factor SPT15 play a crucial role in yeast ethanol tolerance [6]. Watanabe *et al.* also reported that general stress-induced genes are under the control of a cis-acting factor called the stress response element (STRE) [7]. Further studies directly compared the transcriptomes of stressed and non-stressed *S. cerevisiae* during short-term sub-lethal ethanol exposure [8-12] also showed that many genes were differentially expressed in the response to ethanol, which related to cell energetics, transport mechanisms, cell surface interactions, lipid metabolism, general stress response, trehalose metabolism, protein destination, ionic homoeostasis and an increase in the expression of many glycolysis and TCA cycle-associated genes, etc. [3]. Ogawa *et al.* identified 271 genes with increased expression during ethanol stress of the ethanol tolerant mutant SR4-3 using microarray technology [13]. Yoshikawa *et al.* also found 359 ethanol-specific genes by a comprehensive phenotypic analysis under ethanol stress in a collection of yeast strains with a single gene deletion [14].

Compared with *S. cerevisiae*, there are relatively fewer studies regarding ethanol tolerance in bacteria. Gram-negative bacteria usually display an ethanol sensitive phenotype. In *E. coli*, ethanol act as a inhibitor of growth and metabolism, which changes its physical characteristics of cell membrane [15-17]. However, adaptive changes including membrane fatty acid composition, biosynthesis of fatty acid, lipids, peptidoglycan, and outer membrane proteins have reported in response to ethanol stress [17]. For example, the ethanol tolerant mutants (LY01, LY02, and LY03) derivated from engineered *E. coli* KO11 by laboratory adaptive evolution method showed 50% survival rate when exposure to 10% ethanol [18].

Ethanol is also act as a inhibitor of cell growth and metabolism in *Z. mobilis*, thus resulting in decrease in the rate of sugar conversion to ethanol [2]. Previous studies indicated that the lipid composition of *Z. mobilis* may represent an evolutionary adaptation for survival in the presence of ethanol [19]. Further research on protein pattern found that differential expression of related proteins are involved in ethanol-shocked responses [20,21]. However, no other studies have examined the response of *Z. mobilis* to ethanol stresses on genomic level. The first genome sequence for *Z. mobilis* ZM4 suggested that a sigma factor (σE, ZMO4104) may play an important role in resisting ethanol stress [22,23]. As a candidate ethanologenic microorganism for converting cellulosic biomass into ethanol or other valuable chemicals, *Z. mobilis* showed many desirable industrial characteristics for its special Entner-Doudoroff pathway [23]. Different engineered *Z. mobilis* strains have also been successfully constructed [24-27] to convert cellulosic biomass into ethanol. Importantly, the complete genome sequence of different *Z. mobilis* strains (such as ZM4, NCIMB11163, 29192 and 10988, etc.) have been reported since 2005 [22,28-30]. However, the physiological basis and genetic mechanisms involved in ethanol tolerance for *Z. mobilis* are poorly understood. In order to develop new tolerant strains, the mechanisms of *Z. mobilis* in response to ethanol need to be examined and understood, which will provide new insight into tolerance mechanisms and aid future metabolic engineering and synthetic biology in ethanologenic strain improvement. With the completed genome from different *Z. mobilis* strains in hand, comparative genomics or global expression analysis should reveal ways to improve the performance of *Z. mobilis*, and more approaches to strain improvement will certainly be indentified in the future [23].

In this study, microarray technology was used to investigate the expression profiling of the ethanologenic *Z. mobilis* in response to ethanol stress. The results showed 127 genes were expressed up- or down-regulated. These data will help us to understand the molecular mechanisms and provide a global insight into strain improvement by metabolic engineering or synthetic biology.

## Results and discussion

### Profiling of cell growth and glucose utilization under ethanol stress

**Figure 1:**
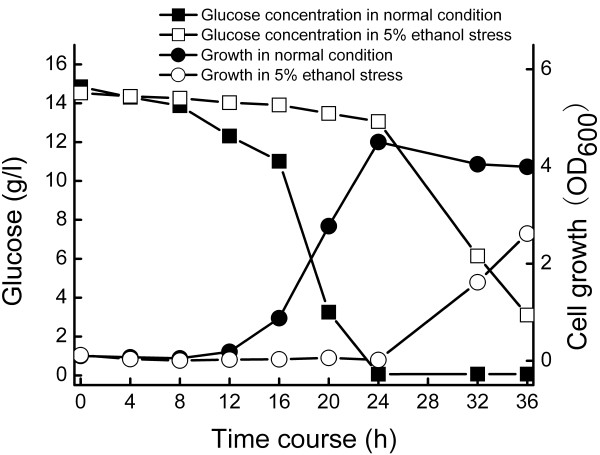
***Z. mobilis *fermentations under normal and ethanol stress conditions.** The data come from mean values of triplicate experiments.

The presence of 5.0% ethanol in the medium led to negative impacts on cell growth, glucose consumption of *Z. mobilis* ZM4 (Figure 1). In the ethanol untreated culture, maximal cell density (OD_600_) reached to 4.5 after approximately 24 h post-inoculation, while the time of *Z. mobilis* needed to reach its highest cell density of 2.62 (OD_600_) delayed until 36 h after initial inoculation under ethanol stress conditions. *Z. mobilis* also consumed glucose more slowly under ethanol stress conditions, nearly 90% of the initial glucose remaining after 24 h incubation. In opposite, almost of the glucose has been utilized at this time point under normal conditions. When *Z. mobilis* growth reached its peak after 36 h under stress conditions, 22% of the glucose also remained in the culture (Figure 1).

### Transcriptome comparision of Z. mobilis under normal and ethanol stress conditions

Seo *et al.* describe the *Z. mobilis* ZM4 (ATCC31821) genome as consisting of a single chromosome and plasmids [22], which we utilized for probe design and microarray fabrication. Therefore, the array data in the present study may fully represent the differences between furfural stress and normal conditions since these plasmid DNA sequences were available. We used a multiplex array format with an average probe length of 36 nucleotides and were able to detect significantly differentially expressed genes.

**Figure 2:**
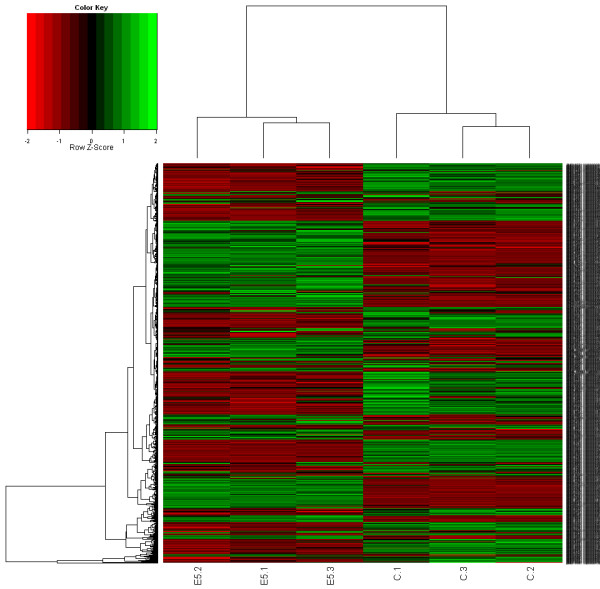
**Hierarchical cluster analysis of significantly differentially expressed ZM4 genes for normal and ethanol stress condition at 24 h.** Gene expression values were clustered based on their log_2_ based expression values. Negative numbers (colored red) indicates less relative gene expression under normal condition, and positive numbers (colored green) indicate greater relative gene expression under ethanol stress.

Based on the genome data of *Z. mobilis*[22], 1800 gene fragments were amplified by PCR and spotted onto the glass slide. With the sophisticated microarray, the global transcriptional response of *Z. mobilis* ZM4 to ethanol stress was examined at 24 h post-inoculation under normal (media with no ethanol) and stress conditions (media with 5% ethanol). Of the 1,800 genes examined by microarray analysis, 127 genes (7% of the total number of open reading frames represented on the array) were identified as being significantly up- or down-regulated (fold change*≥*2.0, *P*≤0.05) during ethanol stress condition. Eighty nine genes were up-regulated after 24 h post-inoculation under ethanol stress condition and 38 genes were down-regulated (Figure 2 and Additional file 1: S1, Table S1 and S2). Table 1 provides a summary of the percentage of differentially expressed genes grouped by functional categories according to TIGR’s annotation of the *Z. mobilis* genome [22]. We have also deposited the entire microarray data at Gene Expression Omnibus (GEO, http://www.ncbi.nlm.nih.gov/geo/) database with the accession number of GSE39558 so interested parties can conduct their analyses (Please see Additional file 2). Approximately 34% of the genes down-regulated in the presence of ethanol were related to metabolism. In the presence of ethanol, about 62% of the genes related to regulation, cell processes, transport, and unknown function showed greater expression as compared to normal conditions. Nearly 24% of the genes including plasmid encoding genes showing greater expression under stress condition. 

**Table 1:** **Number of differentially expressed genes under ethanol stress according to the *Z. mobilis *genome database**

**COG category**	**Up-regulated genes**	**Down-regulated genes**
Energy production and conversion	1	5
Cell cycle control	0	1
Amino acid transport and metabolism	2	3
Nucleotide transport and metabolism;	2	0
Carbohydrate transport and metabolism	2	2
Coenzyme transport and metabolism	4	1
Translation	2	1
Transcription	1	2
Replication, recombination and repair	4	0
Cell wall/membrane biogenesis	4	0
Cell motility	0	2
Posttranslational modification, protein turnover, chaperones	1	1
Inorganic ion transport and metabolism	2	3
General function prediction only	7	4
Function unknown	4	3
Signal transduction mechanisms	4	0
Intracellular trafficking and secretion	2	0
Defense mechanisms	1	0
not in COGs	16	5
plasmid encoding genes.	30	5
Total	89	38

To confirm the microarray results, nighteen genes involving in metabolism, information transfer, plasmid encoding genes and hypothetical proteins were chosen for the qPCR analysis (Table 2). The data showed that qPCR was more sensitive with greater differences in comparison to the microarray results (Additional file 1: Figure S2), which was in keeping with previous reports [31,32]. On the other hand, genes involving in Entner-Doudoroff (ED) and pyruvate pathways were also chosen for the qPCR analysis, which showed the same results as microarray (data not shown). 

**Table 2:** Primers pairs used for q-PCR analysis with target gene information

**Primary Locus**	**Gene**	**Function**	**Forward primer (5**^**′**^**to 3**^**′**^**)**	**Reverse primer (5**^**′**^**to 3**^**′**^**)**	**Product size (bp)**	^a^**Array**	^b^**qPCR**
ZMO0216		peptidase M23	GACATCACTGGCTTCTAA	GCTGGTTCAAGACGATAT	104	1.1	2.6
ZMO0265		hypothetical protein	TAAACAGCAGATGACCTT	ATATTGGACCGATTGGAA	100	2.3	2.3
ZMO0375		levansucrase	TTATGCGGATAGTGAAGG	ACGGAAATTCCAGAGATTA	115	3.0	7.2
ZMO0546		sulphate transporter	TGTCCTGACTCATAATCT	CGCTTATTCTCTTCATCA	120	3	4.8
ZMO0557		hypothetical protein	AGATTATCAGGACTGGAA	TAACATTATCAGCATCGT	113	1.5	3.1
ZMO1417		DEAD/DEAH box helicase domain-containing protein	TTATTGCCAATGACGAAC	TTTTCCATGACAAAGTTTTC	100	1.5	3.1
ZMO1425		thiamine monophosphate synthase	TCATTATCGCTTGCCCTTCA	GAGCCGAATCAGCCAGAA	101	1.1	4.9
ZMO1802		hypothetical protein	TGCTTATGCAGTGTTTGG	TCAGGAAGGTGTAGAGAC	94	1.1	3.5
ZMO1804		amino acid permease-associated domain-containing protein	TTTATGGATTTGATACTGTC	CGCTACACCAATATAGAT	119	1.4	3.71
ZZM4_0013		P2 GpU family protein	GTCACATCCATAGTAGAA	TTATTGTATTGTCGTCATC	106	1.7	4.3
ZZM4_0036		protein of unknown function DUF264	CCAGAATAGTGAAGAAGG	ATCAAGACCTCTAAGTTG	109	1.4	1.13
pzmob1_p05		hypothetical protein	TTCCAATCGGTTCAATTAGT	CAGCCATAGTATCGGTAAG	100	2.11	4.86
pzmob1_p07		hypothetical protein	ATGCTGCTTGGTTTGTTA	GTCATCACAATAGGTAGTCT	107	2.66	4.09
ZMO1063	*pspA*	phage shock protein A	GCCTTATCAGCGATTTATC	GCTTCATTCAACTTATTCTG	100	1.1	2.5
ZMO1064	*pspB*	phage shock protein B	TTAGTCTGCCTTATTCTG	CTCATAAAGCTCTTCAATC	120	1.3	2.0
ZMO1065	*pspC*	phage shock protein C	AAACCGTTTTCGTGATAT	CGCAAATTATCTATTTCCTT	96	1.2	3.0
ZMO0057		phage protein	GATAAAGCGACATTAAAGG	TTCATCACCCAGTATTTC	111	−1.2	−3.21
ZMO0062		aldo/keto reductase	CAACCCGAATATAATCTTTA	AAGCCAGACTGTAATAAG	103	−1.52	−4.7
ZMO1851		flavodoxin FldA	AAATTGACTGGGAGGATA	GACGAAAGAATCTGGATAA	110	−1.93	−4.7
**Endogenous control**							
ZMOr009	*rrsA*	16s RNA	TCAACTATAGACCAGTAAGT	AGAACATAGAAGAGGTAAGT	101		

^a^Array: the log_2_ based microarray ratio of the gene expression (ethanol stress/normal).

^b^qPCR: the log_2_ based qPCR ratio of the gene expression (ethanol stress/normal).

### Transcripts of Entner-Doudoroff (ED) pathway

As expected, 23 Entner-Doudoroff (ED) pathway mRNAs such as *glk*, *zwf*, *pgl*, *pgk* and *eno,* as well as ethanol fermentation-related genes like *pdc* and *adh*B were shown to be less abundant under stress conditions, but at levels not considered significant. Only two genes of ED and pyruvate biosunethetic pathway were showed significantly down-regulated during ethanol stress (1.5-fold change for *gnl*, and 1.1-fold change for *gntK*, Table 3), which may help us to elucidate its negative effects on cell growth or glucose consumption during ethanol stress. Actually, previous studies have hypothesized that this inhibition is due to direct action of ethanol on key enzymes of glycolysis and ethanol production involving feedback inhibition or enzyme inactivation. However, in vitro studies of these enzymes in *Z. mobilis* and *S. cerevisiae* do not fully support this hypothesis. Further research indicated that inhibition of fermentation by ethanol appears to result from increased leakage through the plasma membrane, allowing loss of cofactors and coenzymes, and also coupled with possible additional leakage of intermediary metabolites en route to ethanol formation [2]. On the other hand, *ldh*A also showed nearly significantly down-regulated during ethanol stress condition, which may be lead to decrease lactic acid formation (about 50%) and improve the efficiency of glucose utilization under ethanol stress. 

**Table 3:** Transcripts of Entner-Doudoroff (ED) pathway under ethanol stress

**Primary Locus**	**Gene**	**Fold change in array experiment**
ZMO0366	glf	0.92
ZMO0369	glk (glucokinase)	0.86
ZMO0367	zwf	0.79
ZMO1649	gnl	0.34
ZMO1757	gntK	0.48
ZMO1478	pgl	0.67
ZMO0368	edd	0.89
ZMO0997	eda	0.88
ZMO0177	gap	0.97
ZMO0178	pgk	0.8
ZMO1240	gpm	0.91
ZMO1608	eno	0.87
ZMO0152	pyk	0.87
ZMO1496	Ppc	0.64
ZMO1237	ldhA	0.54
ZMO1360	pdc	0.76
ZMO1596	AdhB	0.89
ZMO0544	CitC	0.91
ZMO0487	Citrate lyase	0.78
ZMO0569	SdhC	0.95
ZMO0705	BudB	1.03
ZMO1955	YqkJ	0.91
ZMO1307	FumA	0.76
ZMO1963	GltA	0.94

### Induction and repression of cell envelope components under ethanol stress

There are 32 ORFs (ZMO0602-ZMO0652: *flgABCDEFGHIJKL, flhAB, fliDEFGHIKLMNPQRS, motAB)* encoding flagellar structure proteins, motor proteins and biosynthesis proteins in *Z. mobilis*. As expected, two genes involving in cell motility showed down-regulated under ethanol stress, such as ZMO0613 (*flgC*, 1.0-fold) and ZMO0614 (*flgB*, 1.3-fold). Most flagellar-related genes, such as ZMO0604 (*flgL*), ZMO0605, ZMO0607, ZMO0608 (*flgH*), ZMO0609, ZMO0610, ZMO0611, ZMO0612, ZMO0619, ZMO0624 (*flhA*), ZMO0632 (*fliE*), ZMO0634, ZMO0635 (*fliG*), ZMO0642, ZMO0643, ZMO0648, ZMO0649, ZMO0651 and ZMO0652 showed also down-regulated under ethanol stress condition.

However, the transcripts encoding MltA domain-containing protein (ZMO2023), cell wall hydrolase *SleB* (ZMO0448), peptidase M23 (ZMO0216) and organic solvent tolerance protein *ostA* (ZMO1311) were shown to be more abundant under stress condition by microarray analysis (see Additional file 1: Table S1). However, other cell wall/membrane biogenesis-related genes, such as ZMO0624, ZMO0641, ZMO0643, ZMO0644, ZMO0647 and ZMO0650 showed less abundant under stress condition by microarray analysis (Data not shown). This suggests that *Z. mobilis* have a adaptive mechanism in response to ethanol stress, which in accordance with previous studies (*Z. mobilis* could tolerant 13% ethanol) [17].

Especially, gene enconding putative organic solvent tolerance protein *ostA* (ZMO1311) showed more abundant under ethanol stress condition, which was in keeping with our previous reports about furfural stress. Further work may be performed by overexpression of this gene to get more tolerant *Z. mobilis* strains for producing valuable chemicals.

### Transcripts of respiratory chain genes

It was reported that Z. mobilis has a *respiratory* electron transport chain and there are 25 respiratory chain genes. Twelve respiratory chain genes were shown to be more abundant under stress condition, including putative Fe-S oxidoreductase (ZMO0022, 1.09-fold), cytochrome bd-type quinol oxidase subunits 1(ZMO1571, 1.52-fold), cytochrome bd-type quinol oxidase subunits 2 (ZMO1572, 1.09-fold), Fe-S-cluster redox enzyme (ZMO1032, 1.04-fold), cytochrome *c*-type biogenesis protein (ZMO1255, ZMO1256, 1.07-fold), ubiquinone biosynthesis protein (ZMO1189, 1.17-fold; ZMO1669, 1.89-fold), nitroreductase (ZMO0678, 2.53-fold), NADH:ubiquinone oxidoreductase complex (ZMO1812, 1.10-fold; ZMO1813, *rnfB*, 1.66-fold; ZMO1814, *rnfA*, 1.47-fold). Interestingly, transcripts of the putative respiratory gene *rnfA* and *rnfB*, were also illustrated to express more greatly (1.9-fold) under furfural stress condition in our previous sutdies [32].

Other 13 respiratory chain genes were shown to be down- regulated (0.6-0.9 fold) in the presence of 5% ethanol after 24 h incubation, including oxidoreductase gene (ZMO1844, 0.51-fold), cytochrome *b* (ZMO0957), cytochrome *c*1 (ZMO0958), cytochrome *c*-type biogenesis proteins (ZMO1252-1254), electron transfer flavoprotein (ZMO1479 and ZMO1480), NADH dehydrogenase (*ndh*, ZMO1113), NADH:flavin oxidoreductase (ZMO1885), NADH:ubiquinone oxidoreductase complex (ZMO1809-ZMO1811), fumarate reductase (ZMO0569). Hayashi *et al.* previously isolated respiratory-deficient mutant (RDM) strains of *Z. mobilis*, which exhibited higher growth and enhanced ethanol productivity under aerobic conditions. Nucleotide sequence analysis revealed that all NADH dehydrogenase-deficient strains were mutated within the *ndh* gene [33]. An upregulation of several thiol-dependent oxidative stress-protective systems was also observed in ndh mutant under aerobically growing [34]. Taken together, knock out *ndh* gene may be improved *Z. mobilis* tolerance.

### Transcripts of universal stress response gene under ethanol stress

*Z. mobilis* contains many ORFs related-to-stress shock-responsive molecular chaperone complex, such as DnaK (ZMO0660), DnaJ (ZMO0661, ZMO1545, ZMO1545, ZMO1069) and GrpE (ZMO0016) of the HSP-70 chaperone complex, GroES-GroEL (ZMO1928 and ZMO1929), HSP-33 (ZMO0410), ZMO0426, ZMO0427, ZMO0949 and ZMO1424, etc. However, these universal stress genes were not affected significantly under ethanol stress (Data not shown).

On the other hand, sigma factors which are responsible for stress tolerance in *E. coli* were also showed higher differentialy expressed in *Z. mobilis*, such as sigma-E (σE, ZMO1404, 1.3-fold), σ70 (*rpo*D, ZMO1623, 1.7-fold), σ54 (*rpo*N, ZMO0274, 1.2-fold) and σ28 (*fli*A, ZMO0626, 1.4-fold). Seo *et al.* also supposed that sigma-E plays a key role in resisting high ethanol conditions in *Z. mobilis*, which was in keeping with our current study about ethanol stress. Further work may be performed by global tanscriptional metabolic engineering (gTME) [35] via σE to improve tolerance in *Z. mobilis*.

### Terpenoid biosynthesis under ethanol stress

Hopanoids are a class of pentacyclic triterpenoid lipids that occur in a wide range of Gram-negative and Gram-positive bacteria. Recently study further indicated that hopanoids play an important role in maintaining membrane integrity and pH homeostasis in *Rhodopseudomonas palustris* TIE-1 [36]. There are five open reading frames (designated as *hpnA-E*) in a close arrangement with *shc* gene (ZMO0872, the squalene-hopene cyclase, *hpnF*) [37]. In this study, the genes such as *hpnC* (ZMO0869) and *hpnD* (ZMO0870) involving in hopanoid biosynthesis pathway were shown to be down-regulated (0.8-fold) in the presence of 5% ethanol after 24 h incubation. However**,***hpnA* (ZMO0867), *hpnB* (ZMO0868) and *shc* were shown to be up-regulated (nearly 1.4-fold) under the same condition.

Other terpenoid biosynthesis related gene, such as *ispB* (ZMO0564), *ispH* (ZMO0875), *ispE* (ZMO1182), *hpnH* (ZMO0874), *hpnI* (ZMO0972), *hpnJ* (ZMO0973), *hpnK* (ZMO0974) and *dxr* (ZMO1150) exhibited a up-regulated expression pattern. However, transcripts such as *deoD* (ZMO0873), *dxs* (ZMO1234 and ZMO1598), *ispDF* (ZMO1128), *ispG* (ZMO0180), *ispA* (ZMO0855), *uppS* (ZMO1152), *hpnM* (ZMO0876) and *hpnN* (ZMO1599) showed a down-regulated expression pattern.

Previous studies indicated that *Z. mobilis* have the highest total hopanoid content (30 mg/g DCW, dry cell weight) among all bacterias, which lead to more tolerant by increasing of the hopanoid content [38,39]. However, another research in *Z. mobilis* ATCC29191 showed that addition of ethanol to the media caused complex changes in the levels of hopanoids and none of the hopanoid lipid classes increased significantly [40]. Carey and Ingram *et al.* also showed that vaccenic acid represents over 75% of the acyl chains in the polar membrane lipids in *Z. mobilis*. Ethanol had no major effect on the fatty acid composition of *Z. mobilis*, which showed a high constitutive expressed mode even under stress. However, ethanol caused a decrease in phosphatidylethanolamine and phosphatidylglycerol, and an increase in cardiolipin and phosphatidylcholine. Ethanol also caused a dose-dependent reduction in the lipid-to-protein ratios of crude membranes. These results were in keeping with our array experiment.

Taken together, these data suggest that ethanol has a negative effect on terpenoid biosynthesis, and then may damage the cell membrane of *Z. mobilis*. However, the dynamic changes in lipid composition may represent an evolutionary adaptation for survival in the presence of ethanol [19].

### Transcripts of gene related to DNA replication, recombination and repair

There are 82 ORFs related to DNA replication, recombination and repair in Z. mobilis genome [22]. Five genes related to DNA replication, recombination and repair were revealed as being up-regulated in the presence of 5% ethanol after 24 h incubation, such as *dnaA* (ZMO1356), DNA repair protein *radC* (ZMO1426), ZMO1484, ZMO1417 and *ung* (ZMO1648) (see Additional file 1: Table S1). Fourty-one genes also showed higer expressed from 1.02~1.99-fold change in this array, such as *topA* (ZMO1193), ZMO1401, *dnaZX* (ZMO086), ZMO1582, *intZ* (ZMO1930), ZMO0888, *holA* (ZMO1433), *xerD* (ZMO0598), *mutS* (ZMO1907), ZMO1194, *mutM* (ZMO1187), *parC* (ZMO1054), *recF* (ZMO1584), ZMO1185, *recR* (ZMO0812). However, transcripts encoding the others genes related to DNA replication, recombination and repair were found to be less abundant (0.5~0.98 fold change) under ethanol stress, such as *mboA* (ZMO0575, 0.54-fold), *dnaQ* (ZMO0039, 0.53-fold), *ihfB* (ZMO1801, 0.51-fold) and ZMO1989.

### Transcriptional regulation under ethanol stress

Fifty-four transcriptional activators and repressors were identified in *Z. mobilis* genome [22]. In this study, 33 transcriptional regulators were down-regulated under ethanol stress. However, only 3 transcriptional regulators including MarR family transcriptional regulator ZMO0054, 1.9-fold) , XRE family transcriptional regulator (ZMO2033, 1.5-fold) and HxlR family transcriptional regulator (ZMO1697, 1.8-fold) showed significant differentialy expressed (see Additional file 1: Table S2).

Transcriptional regulator ZMO1107 (Lrp-like, sharing 40% identity to *E. coli* global regulator Lrp) and ZMO0347 (sharing 60% identity to *E. coli* global regulator *Hfq*) [31], which also showed less abundant under ethanol stress. Previous studies revealed that *E. coli* global regulator *Lrp* affected the expression of at least 10% of all *E. coli* genes [41,42]. Hfq is an RNA-binding protein that is common to diverse bacterial lineages and has key roles in the control of gene expression. Hfq also affected the translation and turnover rates of specific transcripts, which contributes to complex post-transcriptional networks [31,43]. Hfq could also associated with *E. coli* motor protein Rho to mediate transcription antitermination via a novel transcription regulatory mechanism [44]. Recently studies also indicated that *Hfq* may play an important role in sRNA network control [45,46]. Yang *et al.* also showed that *Z. mobilis hfq* contributes to tolerance against multiple lignocellulosic pretreatment inhibitors [47], which provide a fundamental example for further studies or industrial strain development in the future.

Other transcriptional regulators were shown to be more abundant under ethanol stress, such as TetR family transcriptional regulator (ZMO0281, ZMO1547), LysR family transcriptional regulator (ZMO0774) and RpiR family transcriptional regulator (ZMO0190).

Two phage shock protein B and C (ZMO1064, *pspB* and ZMO1065, *pspC*) were shown to be higher differentially expressed (see Additional file 1: Table S1). Anoter phage shock protein A (ZMO1063, *psp*A, 1.64-fold) was also showed higer differentially expressed under ethanol stress. These results is in keeping with our previous study on furfural stress [32], which may be indicated that Psp play an important role in response to different stress. There are four phage shock proteins in the ZM4 genome (ZMO1061, ZMO1063, ZMO1064, ZMO1065), which may encode and consist of a *psp* regulon combined with a hypothetical protein (ZMO1062) [22]. We deduce that there is a *psp-*like regulon in response to stress in *Z. mobilis*. Actually, the Phage shock protein response was originally discovered in P. Model’s laboratory at the Rockefeller University with studying filamentous phage f1 infection of *E. coli*[48]. Now, *Psp* protein is found out across Gram-positive bacteria, Gram-negative bacteria, archaea to plants, and might perceive cell membrane stress and signal to the transcription apparatus by using an ATP hydrolytic transcription activator to produce effector proteins to overcome different stress [49], such as phage infection, secretin production, blockage of protein export or fatty acid/phospholipid biosynthesis, organic solvents, heat, osmotic, pH, etc. [50]. However, function of psp regulon is still unclear, which should be elucidated in the future on molecular level.

### Transcripts of gene related to transport systems

In this study, 12 ORFs related to carbohydrate, amino acid, nucleotide, coenzyme and inorganic ion transport and metabolism showed significant expressed under ethanol stress, such as ZMO1180, ZMO2018, *hutG* (ZMO1395), ZMO1804, *nrdD* (ZMO1025), *yfeJ* (ZMO1855), ZMO1522, *thiE* (ZMO1425), *folK* (ZMO1647), *ssuC* (ZMO1262) and ZMO0546 (see Additional file 1: Table S1). However, 3 ORFs related carbohydrate and coenzyme transport and metabolism showed a down-regulated expression pattern, such as *gnl* (ZMO1649), *gntK* (ZMO1757) and ZMO0899 (see Additional file 1: Table S2).

### Induction of plasmid encoding genes under ethanol stress

Interestingly, 30 genes from ZM4 plasmids were shown to be more abundant (1.0-2.7 fold, based on log_2_ system) under ethanol stress condition. However, most of these genes encode hypothetical proteins, such as pzmob1_p05, pzmob1_p06, pzmob1_p07, ZZM4_0013, ZZM4_0026, ZZM4_0027, ZZM4_0114. Especially, pzmob1_p05, pzmob1_p18 and pzmob1_p19 should the same profling between furfural and etanol stress [32].

Five plasmid encoding genes showed less abundant under the same condition (1–1.4 fold, based on log_2_ system), such as ZZM4_0002, ZZM4_0006, ZZM4_0121, ZZM4_0154 and ZZM4_0156 (see Additional file 1: Table S1 and Table S2). However, pzmob1_p06 and pzmob1_p07 showed more abundant under furfural stress, and less abundant during 5% ethanol condition. It may be indicated that different plasmid encoding genes are responsible to different stress. Furthermore, the function of plasmid encoding genes are still unclear, and further work should be focing on these genes.

## Conclusion

In this study, we successfully identified genes involved in ethanol tolerance by microarray analysis. These genes were classified as being involved in a wide range of cellular processes including carbohydrate metabolism, cell wall/membrane biogenesis, respiratory chain, terpenoid biosynthesis, DNA replication, DNA recombination, DNA repair, transport, transcriptional regulation, some universal stress response, etc. Our study indicated that ethanol tolerance in *Z. mobilis* is affected by various complicated processes that take place on both the molecular and the cellular level, and that membrane might play important roles in response to ethanol. Although the molecular mechanism involved in tolerance and adaptation of ethanologenic strains to ethanol is still unclear, this research has provided insights into molecular response to ethanol in *Z. mobilis*. These data will also be helpful to construct more ethanol resistant strains for cellulosic ethanol production in the future.

## Material and methods

### Bacterial strains and fermentation conditions

*Z. mobilis* ZM4 (ATCC31821) was cultured in Rich media (RM) [51] at 30°C without shaking. Cultures were maintained on glucose agar (20.0 g/l glucose, 10.0 g/l yeast extract and 15.0 g/l agar). Organism was subcultured to fresh inoculum media for 24 h at 30°C before being inoculated into the fermentation medium. Inoculum medium (g/l) consisted of 10.0 g yeast extract, 1.0 g MgCl_2_, 1.0 g (NH_4_)_2_SO_4_, 1.0 g KH_2_PO_4_, 20.0 g glucose. The final concentration of ethanol was set up at 5% (v/v) for the study of the response of ethanol stress in *Z. mobilis*. The optical density was measured with a spectrophotometer at 600 nm with an initial OD_600_ of 0.05 when the inoculum was added to each flask (with or without 5% ethanol).

### Cell growth and glucose analysis

Cell growth was determined by monitoring the optical density at 600 nm by using Multi Scanner Spectrometer (Thermo Inc.) at 4-h intervals. Fermentation supernatant was prepared by passing through 0.2 μm membrane (Millipore) and used to determine the concentrations of glucose. Ions Chromatography (Switzerland, Metrohm Bio-Scan 871) was applied to measure the concentration of glucose with sodium hydroxide (0.1 M) as mobile phase at a flow rate of 1.0 ml/min as described previously [32].

### RNA isolation, fluorescein-labeled Cdna and microarray

Total RNA was isolated essentially described previously [32,52]. The RNA quality was assessed by formaldehyde agarose gel electrophoresis and quantitated at OD_260_ and OD_280_ by spectrophotometer, respectively. The purified RNA from each sample was used as the template to generate cDNAs while labeled with either Cy3-dUTP or Cy5-dUTP (CapitalBio) in a duplicate set.

*Z. mobilis* microarrays were constructed by CapitalBio Corporation (Beijing, China) using coding sequences predicted by The Institute for Genomic Research (TIGR, http://www.tigr.org/). Microarray hybridization, washing, scanning and data analysis were carried out according to the NimbleGen’s Expression user’s guide. Gene expression analysis was performed using six independent microarray experiments (two dye reversal reactions × three biological replicates) with each microarray containing one to two probes per predicted coding sequence each.

Hierarchical clustering and comparison analysis were performed by Cluster 3.0 and SAM 3.02 software, respectively. Sgnificantly differentially expressed genes were determined with a selection threshold of false discovery rate, FDR<5% and fold change*≥*2.0 (significant induction) or ≤0.5 (significant repression). Raw data was log_2_ transformed and imported.

### Quantitative-PCR (qPCR) analysis

Real-time quantitative-PCR (RT-PCR) was performed to verify the microarray expression results. The purified RNA samples were reverse-transcribed by using the Protoscript First Strand cDNA Synthesis Kit (MBI, Fermentas Inc.) as described in the manufacturer’s protocol. Based on microarray hybridizations, nighteen genes representing different functional categories and a range of gene expression values were selected for qPCR assay (iQ5 Real-Time PCR System, Bio-Rad). Optimized 18–20 bp primers for qPCR analysis listed in Table 1, which were designed to amplify 90–120 bp of the target genes. First-strand cDNA was synthesized using a cDNA synthesis kit (MBI, Fermentas Inc.). PCR conditions were 10 min at 94°C, followed by 40 cycles of heating at 94°C for 20 s and 60°C for 30 s, and final extension at 72°C for 5 min. PCR amplification was detected by SYBR fluorescence dye (Takara). The *rrsA* gene (ZMOr009), encoding the 16S ribosomal RNA gene, served as an endogenous control to normalize for differences in total RNA quantity.

## Abbreviations

ED: Entner-Doudoroff; Psp: Phage shock protein.

## Competing interests

The authors declare that they have no competing interests.

## Authors’ contributions

He Ming-xiong carried out all of the experiments, participated in the study design and wrote the manuscript. The other authors participated in the design of the study and helped in manuscript writing. All authors read and approved the final manuscript.

## Supplementary Material

Additional file 1 **Table S1.** Ethanol stress up-regulated genes after 24 h post inoculation. Table S2. Ethanol stress down-regulated genes after 24 h post inoculation. **Figure S1.** Volcano plot result from JMP Genomics analysis showing significantly differentially expressed genes under ethanol stress condition. Green dots indicate down-regulated genes and red dots indicate up-regulated genes. Black colored dots were not considered significantly differentially expressed. The X axis shows the difference values between ethanol stress and normal conditions based on a log_2_ scale. The Y axis shows statistical significance values for expression values, based on a -log10 *p*-value. The grey line shows the statistical significance cut-off used in this study. **Figure S2.** Comparison of stationary growth phase gene expression measurements by microarray and qPCR. The gene expression ratios for wild-type *Z. mobilis* ZM4 under ethanol and normal conditions after 24 h fermentation were log transformed in base 2. The microarray ratio values were plotted against the qPCR values. Comparison of the two methods indicated a high level of concordance (R = 0.94). Click here for file

Additional file 2 http://www.ncbi.nlm.nih.gov/geo/query/acc.cgi?token=vrsxbkcsaoiuone&acc=GSE39558. Click here for file
