# Supplementary material for: Transcriptome profiling of Zymomonas mobilis under ethanol stress
Source: Biotechnol Biofuels. 2012 Oct 11;5:75. doi: 10.1186/1754-6834-5-75 (PMC3495753; doi:10.1186/1754-6834-5-75)
Supplement: Additional file 1 — Table S1. Ethanol stress up-regulated genes after 24 h post inoculation. Table S2. Ethanol stress down-regulated genes after 24 h post inoculation. Figure S1. Volcano plot result from JMP Genomics analysis showing significantly differentially expressed genes under ethanol stress condition. Green dots indicate down-regulated genes and red dots indicate up-regulated genes. Black colored dots were not considered significantly differentially expressed. The X axis shows the difference values between ethanol stress and normal conditions based on a log2 scale. The Y axis shows statistical significance values for expression values, based on a -log10 p-value. The grey line shows the statistical significance cut-off used in this study. Figure S2. Comparison of stationary growth phase gene expression measurements by microarray and qPCR. The gene expression ratios for wild-type Z. mobilis ZM4 under ethanol and normal conditions after 24 h fermentation were log transformed in base 2. The microarray ratio values were plotted against the qPCR values. Comparison of the two methods indicated a high level of concordance (R = 0.94). [file 1754-6834-5-75-S1.doc]

Table S1. Ethanol stress up-regulated genes after 24 h post inoculation.

| **Primary Locus** | **Gene** | **Function** | | **^a^EC** | **^b^Array** | **^c^qPCR** |
| --- | --- | --- | --- | --- | --- | --- |
| **Metabolism** | | | | | | |
| ZMO1180 |  | "carbohydrate kinase, YjeF-like protein" | |  | 1.2 |  |
| ZMO1395 | *hutG* | N-formylglutamate amidohydrolase | |  | 1.2 |  |
| ZMO1804 |  | amino acid permease-associated domain-containing protein | |  | 1.4 | 3.7 |
| ZMO1025 | *nrdD* | anaerobic ribonucleoside triphosphate reductase | |  | 1.1 |  |
| ZMO1855 |  | glutamine amidotransferase | |  | 1.0 |  |
| ZMO1522 |  | TonB-dependent receptor | |  | 1.1 |  |
| ZMO1298 |  | TonB-dependent receptor plug | |  | 1.2 |  |
| ZMO1425 |  | thiamine monophosphate synthase | |  | 1.1 | 4.9 |
| ZMO1647 |  | 2-amino-4-hydroxy-6- hydroxymethyldihydropteridine pyrophosphokinase | |  | 1.6 |  |
| ZMO0678 |  | nitroreductase | |  | 1.3 |  |
| **Information Transfer** | | | | | | |
| ZMO1417 |  | DEAD/DEAH box helicase domain-containing protein | |  | 1.5 | 3.1 |
| ZMO1179 |  | (Uracil-5)-methyltransferase | |  | 1.0 |  |
| ZMO1065 | *pspC* | "phage shock protein C, PspC" | |  | 1.2 | 3.0 |
| ZMO1356 | *dnaA* | chromosomal replication initiation protein | |  | 1.2 |  |
| ZMO1426 |  | DNA repair protein RadC | |  | 1.4 |  |
| ZMO1484 |  | UvrD/REP helicase | |  | 1.5 |  |
| ZMO1648 | *ung* | Uracil-DNA glycosylase superfamily protein | | 3.2.2.- | 2.0 |  |
| ZMO1324 |  | HPr kinase | |  | 2.1 |  |
| ZMO0085 |  | methyl-accepting chemotaxis sensory transducer | |  | 1.4 |  |
| ZMO1487 |  | diguanylate phosphodiesterase | |  | 1.4 |  |
| ZMO1055 |  | diguanylate cyclase/phosphodiesterase | |  | 1.4 |  |
| ZMO1295 |  | organic radical activating-like protein | |  | 1.8 |  |
| ZMO0924 | *secA* | preprotein translocase subunit SecA | |  | 1.0 |  |
| ZMO1183 |  | TPR repeat-containing protein | |  | 1.0 |  |
| **Cell Process** | | | | | | |
| ZMO1311 | *ostA* | organic solvent tolerance protein | |  | 1.2 |  |
| ZMO0216 |  | peptidase M23 | |  | 1.1 | 2.6 |
| ZMO0448 |  | cell wall hydrolase SleB | |  | 1.3 |  |
| ZMO2023 |  | MltA domain-containing protein | |  | 1.7 |  |
| **Transporter** | | | | | | |
| ZMO1029 |  | ABC transporter-like protein | |  | 1.8 |  |
| ZMO1262 | *ssuC* | binding-protein-dependent transport systems inner membrane component | |  | 1.2 |  |
| ZMO0546 |  | sulphate transporter | |  | 3.0 | 4.8 |
| ZMO2018 |  | major facilitator superfamily protein | |  | 1.0 |  |
| **Function Unknown** | | | | | | |
| ZMO0479 |  | | peptidoglycan-binding LysM |  | 1.5 |  |
| ZMO0551 |  | | tRNA pseudouridine synthase B |  | 1.2 |  |
| ZMO0746 |  | | hypothetical protein |  | 1.1 |  |
| ZMO0557 |  | | hypothetical protein |  | 1.5 | 3.1 |
| ZMO0374 |  | | levansucrase |  | 1.6 |  |
| ZMO0375 |  | | levansucrase |  | 1.6 | 7.2 |
| ZMO1064 | *pspB* | | phage shock protein B |  | 1.3 | 2.0 |
| ZMO1473 |  | | hypothetical protein |  | 1.3 |  |
| ZMO1511 |  | | hypothetical protein |  | 2.0 |  |
| ZMO0265 |  | | hypothetical protein |  | 1.2 | 2.3 |
| ZMO0397 |  | | hypothetical protein |  | 1.3 |  |
| ZMO0447 |  | | hypothetical protein |  | 1.1 |  |
| ZMO0849 |  | | Alg9 family protein mannosyltransferase |  | 1.2 |  |
| ZMO1012 |  | | hypothetical protein |  | 1.1 |  |
| ZMO1030 |  | | hypothetical protein |  | 1.2 |  |
| ZMO1045 |  | | phosphate-selective porin O and P |  | 1.2 |  |
| ZMO1802 |  | | hypothetical protein |  | 1.1 |  |
| ZMO1961 |  | | hypothetical protein |  | 1.2 | 3.5 |
| ZMO2030 |  | | hypothetical protein |  | 1.1 |  |
| ZMO1458 |  | | hypothetical protein |  | 1.0 |  |
| ZMO1273 |  | | amidohydrolase 3 |  | 1.3 |  |
| ZMO1413 |  | | ribosomal-protein-alanine acetyltransferase |  | 1.1 |  |
| ZMO0136 |  | | Sel1 domain-containing protein repeat-containing protein |  | 1.3 |  |
| ZMO0137 |  | | Sel1 domain-containing protein repeat-containing protein |  | 1.4 |  |
| ZMO0917 |  | | 2-nitropropane dioxygenase NPD |  | 1.2 |  |
| ZMO1067 |  | | Fe-S metabolism associated SufE |  | 1.0 |  |
| ZMO1882 |  | | membrane protein AbrB duplication |  | 1.0 |  |
| **Plasmid encoding genes** | | | | | | |
| pzmob1_p07 |  | | hypothetical protein |  | 2.7 | 4.1 |
| pzmob1_p06 |  | | hypothetical protein |  | 2.5 |  |
| pzmob1_p05 |  | | hypothetical protein |  | 2.1 | 4.9 |
| ZZM4_0027 |  | | hypothetical protein |  | 2.1 |  |
| ZZM4_0026 |  | | hypothetical protein |  | 2.0 |  |
| ZZM4_0114 |  | | hypothetical protein |  | 1.9 |  |
| ZZM4_0013 |  | | P2 GpU family protein |  | 1.7 | 4.3 |
| ZZM4_0028 |  | | hypothetical protein |  | 1.5 |  |
| ZZM4_0025 |  | | hypothetical protein |  | 1.5 |  |
| ZZM4_0037 |  | | "phage portal protein, PBSX family" |  | 1.4 |  |
| ZZM4_0023 |  | | phage tail protein I |  | 1.4 |  |
| ZZM4_0036 |  | | protein of unknown function DUF264 |  | 1.4 | 1.1 |
| ZZM4_0024 |  | | Baseplate J family protein |  | 1.3 |  |
| pzmob1_p38 |  | | hypothetical protein |  | 1.3 |  |
| ZZM4_0111 |  | | outer membrane autotransporter barrel domain protein |  | 1.2 |  |
| pzmob1_p19 |  | | hypothetical protein |  | 1.2 |  |
| ZZM4_0005 |  | | hypothetical protein |  | 1.2 |  |
| pzmob1_p18 |  | | hypothetical protein |  | 1.2 |  |
| ZZM4_0134 |  | | ABC transporter related protein |  | 1.2 |  |
| ZZM4_0039 |  | | Cobyrinic acid ac-diamide synthase |  | 1.2 |  |
| pzmob1_p16 |  | | hypothetical protein |  | 1.2 |  |
| ZZM4_0014 |  | | "phage tail tape measure protein, TP901 family" |  | 1.2 |  |
| ZZM4_0110 |  | | hypothetical protein |  | 1.1 |  |
| ZZM4_0151 |  | | putative secreted protein |  | 1.1 |  |
| ZZM4_0031 |  | | tail X family protein |  | 1.1 |  |
| ZZM4_0029 |  | | "N-acetylmuramyl-L-alanine amidase, negative regulator of AmpC, AmpD" |  | 1.1 |  |
| ZZM4_0008 |  | | hypothetical protein |  | 1.1 |  |
| ZZM4_0129 |  | | hypothetical protein |  | 1.1 |  |
| pzmob1_p20 |  | | hypothetical protein |  | 1.0 |  |
| pzmob1_p33 |  | | hypothetical protein |  | 1.0 |  |

^a^EC #: Enzyme commission number;

^b^Array: the log_2_ based microarray ratio of the gene expression (ethanol stress/normal);

^c^qPCR: the log_2_ based qPCR ratio of the gene expression (ethanol stress/normal)

Table S2. Ethanol stress down-regulated genes after 24 h post inoculation.

| **Primary Locus** | **Gene** | **Function** | **^a^EC** | **^b^Array** | **^c^qPCR** |
| --- | --- | --- | --- | --- | --- |
| **Metabolism** | |  |  |  |  |
| ZMO0062 |  | aldo/keto reductase |  | -1.5 | -2.2 |
| ZMO0543 |  | aconitate hydratase |  | -1.2 |  |
| ZMO1284 |  | gluconate 2-dehydrogenase |  | -1.2 |  |
| ZMO1696 |  | zinc-binding alcohol dehydrogenase family protein |  | -1.4 |  |
| ZMO1851 |  | flavodoxin FldA |  | -1.9 | -4.7 |
| ZMO1649 | *gnl* | gluconolactonase | 3.1.1.17 | -1.5 |  |
| ZMO1757 |  | thermoresistant glucokinase family carbohydrate kinase |  | -1.1 |  |
| ZMO0899 |  | NAD synthetase |  | -1.1 |  |
| ZMO0734 |  | "3'(2'),5'-bisphosphate nucleotidase" |  | -1.2 |  |
| ZMO1540 |  | FeoA family protein |  | -1.2 |  |
| ZMO0491 |  | peptidase M24 |  | -1.2 |  |
| ZMO1285 |  | glucose-methanol-choline oxidoreductase |  | -1.4 |  |
| ZMO1632 |  | succinyl-diaminopimelate desuccinylase |  | -1.9 |  |
| **Information transfer** | |  |  |  |  |
| ZMO0952 |  | tRNA/rRNA methyltransferase SpoU |  | -1.1 |  |
| ZMO0054 |  | MarR family transcriptional regulator |  | -1.9 |  |
| ZMO1697 |  | HxlR family transcriptional regulator |  | -1.8 |  |
| ZMO0998 |  | peptide methionine sulfoxide reductase |  | -1.5 |  |
| **Cell Process** |  |  |  |  |  |
| ZMO0043 |  | Maf family protein |  | -1.1 |  |
| ZMO0614 | *flgB* | flagellar basal-body rod protein FlgB |  | -1.3 |  |
| ZMO0613 | *flgC* | flagellar basal-body rod protein FlgC |  | -1.0 |  |
| **Transporter** | |  |  |  |  |
| ZMO0978 |  | periplasmic binding protein |  | -1.1 |  |
| **Function unknown** | | |  |  |  |
| ZMO0057 |  | phage protein |  | -1.2 | -3.2 |
| ZMO1633 |  | hypothetical protein |  | -1.2 |  |
| ZMO1671 |  | hypothetical protein |  | -1.2 |  |
| ZMO2033 |  | XRE family transcriptional regulator |  | -1.5 |  |
| ZMO1629 |  | hypothetical protein |  | -1.0 |  |
| ZMO0312 |  | amidohydrolase |  | -1.1 |  |
| ZMO1688 |  | folate-binding protein YgfZ |  | -1.5 |  |
| ZMO1945 |  | PhzF family phenazine biosynthesis protein |  | -1.2 |  |
| ZMO1800 |  | PhzF family phenazine biosynthesis protein |  | -1.0 |  |
| ZMO2034 |  | hypothetical protein |  | -1.3 |  |
| ZMO1850 |  | hypothetical protein |  | -1.2 |  |
| ZMO1289 |  | Transglycosylase-associated protein |  | -1.0 |  |
| **Plasmid encoding genes** | | |  |  |  |
| ZZM4_0002 |  | "addiction module antitoxin, RelB/DinJ family" |  | -1.4 |  |
| ZZM4_0121 |  | hypothetical protein |  | -1.4 |  |
| ZZM4_0156 |  | "addiction module antitoxin, RelB/DinJ family" |  | -1.1 |  |
| ZZM4_0154 |  | Cobyrinic acid ac-diamide synthase |  | -1.1 |  |
| ZZM4_0006 |  | hypothetical protein |  | -1.0 |  |

^a^EC #: Enzyme commission number;

^b^Array: the log_2_ based microarray ratio of the gene expression (ethanol stress/normal);

^c^qPCR: the log_2_ based qPCR ratio of the gene expression (ethanol stress/normal)

**
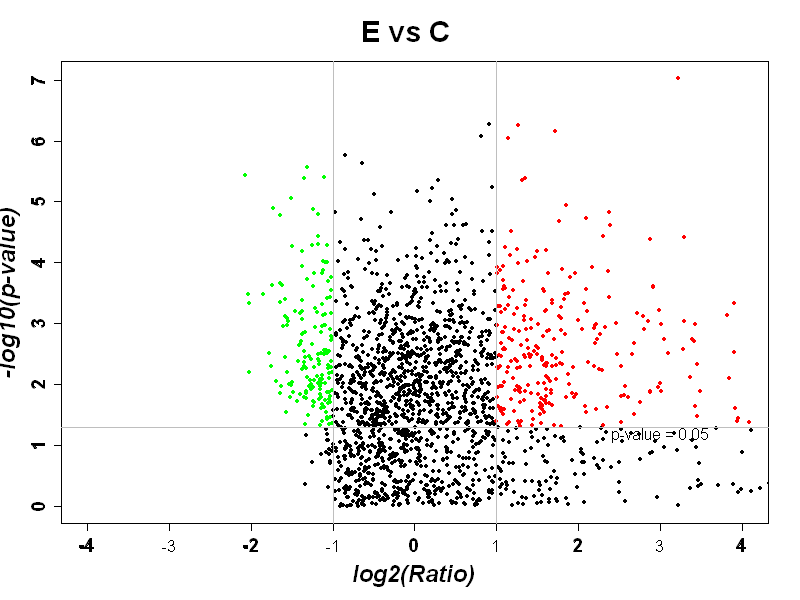
Figure S1. Volcano plot result from JMP Genomics analysis showing significantly differentially expressed genes under ethanol stress condition**. Green dots indicate down-regulated genes and red dots indicate up-regulated genes. Black colored dots were not considered significantly differentially expressed. The X axis shows the difference values between ethanol stress and normal conditions based on a log_2_ scale. The Y axis shows statistical significance values for expression values, based on a -log10 *p*-value. The grey line shows the statistical significance cut-off used in this study.


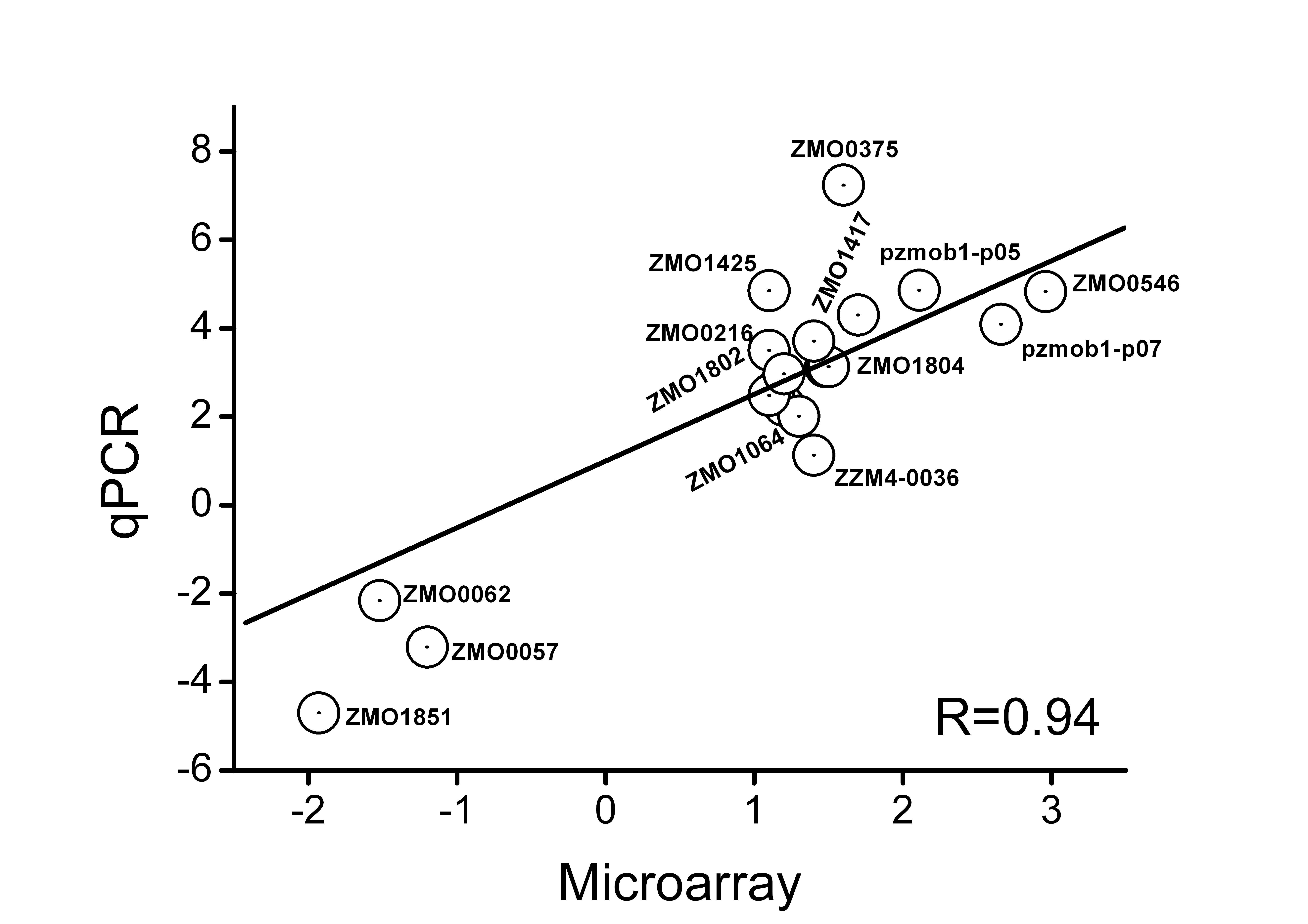


**Figure S2. Comparison of stationary growth phase gene expression measurements by microarray and qPCR.** The gene expression ratios for wild-type *Z.* *mobilis* ZM4 under ethanol and normal conditions after 24 h fermentation were log transformed in base 2. The microarray ratio values were plotted against the qPCR values. Comparison of the two methods indicated a high level of concordance (R = 0.94)
