# Supplementary material for: Transcriptome profiling of Zymomonas mobilis under ethanol stress
Source: Biotechnol Biofuels. 2012 Oct 11;5:75. doi: 10.1186/1754-6834-5-75 (PMC3495753; doi:10.1186/1754-6834-5-75)
Supplement: Additional file 2 — http://www.ncbi.nlm.nih.gov/geo/query/acc.cgi?token=vrsxbkcsaoiuone&acc=GSE39558. [file 1754-6834-5-75-S2.docx]

<http://www.ncbi.nlm.nih.gov/geo/query/acc.cgi?token=vrsxbkcsaoiuone&acc=GSE39558>
